# Retraction: RUNX2 promotes epithelial differentiation of ADSCs and burn wound healing via targeting E-cadherin

**DOI:** 10.18632/oncotarget.28729

**Published:** 2025-05-20

**Authors:** Qiang Li, Han Zhao, Sizhan Xia, Hanxiao Wei, Feifei Chen, Peisheng Jin

**Affiliations:** ^1^Department of Plastic Surgery, Affiliated Hospital of Xuzhou Medical University, Xuzhou, Jiangsu, China; ^2^Jiangsu Center for the Collaboration and Innovation of Cancer Biotherapy, Cancer Institute, Xuzhou Medical University, Xuzhou, Jiangsu, China; ^3^Department of Plastic Surgery, Xuzhou Central Hospital, Xuzhou, Jiangsu, China; ^*^These authors have contributed equally to this work

**This article has been retracted:** Oncotarget has completed its investigation of this paper. It has been determined that numerous images have been manipulated throughout the paper. More specifically, the immunofluorescent (IF) images in Figures 1D, 3H and 4D are overlapping despite the use of different antibodies (CK18 vs E-cadherin vs. RUNX2). Similar overlaps were identified in IF images across Figures 2G, 3H and 3G, as well as Figures 2H, 3G and 4D, where images representing staining with different antibodies at different experimental conditions were overlapped. In Figure 6A, the same image of a mouse appears in the first and last panels for Day 1. The panels in Figure 6C (HE staining) and in Figure 7A (CD31 staining) each contain two overlapping IHC images that are intended to depict different experimental conditions. Given the significant number of duplications and overlaps found within this paper, the editors have concluded that the integrity of the data is substantially compromised, casting doubt on the manuscript’s conclusions. Consequently, the editorial decision has been made to retract this paper. The authors were notified of this decision; however, no response was received.

Original article: Oncotarget. 2018; 9:2646–2659. 2646-2659. https://doi.org/10.18632/oncotarget.23522

